# A chromosome‐scale reference of *Chenopodium watsonii* helps elucidate relationships within the North American A‐genome *Chenopodium* species and with quinoa

**DOI:** 10.1002/tpg2.20349

**Published:** 2023-05-17

**Authors:** Lauren A. Young, Peter Jeffrey Maughan, David E. Jarvis, Spencer P. Hunt, Heather C. Warner, Kristin K. Durrant, Tyler Kohlert, Ramiro N. Curti, Daniel Bertero, Gabrielle A. Filippi, Tereza Pospíšilíková, Karol Krak, Bohumil Mandák, Eric N. Jellen

**Affiliations:** ^1^ Plant and Wildlife Sciences Department Brigham Young University Provo Utah USA; ^2^ Facultad de Ciencias Naturales Universidad Nacional de Salta CCT‐CONICET Salta Argentina; ^3^ Cátedra de Producción Vegetal, Facultad de Agronomía Universidad de Buenos Aires and IFEVA‐CONICET Buenos Aires Argentina; ^4^ Faculty of Environmental Sciences Czech University of Life Sciences Prague Prague Czech Republic; ^5^ Institute of Botany Czech Academy of Sciences Průhonice Czech Republic

## Abstract

Quinoa (*Chenopodium quinoa*), an Andean pseudocereal, attained global popularity beginning in the early 2000s due to its protein quality, glycemic index, and high fiber, vitamin, and mineral contents. Pitseed goosefoot (*Chenopodium berlandieri*), quinoa's North American free‐living sister species, grows on disturbed and sandy substrates across the North America, including saline coastal sands, southwestern deserts, subtropical highlands, the Great Plains, and boreal forests. Together with South American avian goosefoot (*Chenopodium hircinum*) they comprise the American tetraploid goosefoot complex (ATGC). Superimposed on pitseed goosefoot's North American range are approximately 35 AA diploids, most of which are adapted to a diversity of niche environments. We chose to assemble a reference genome for Sonoran A‐genome *Chenopodium watsonii* due to fruit morphological and high (>99.3%) preliminary sequence‐match similarities with quinoa, along with its well‐established taxonomic status. The genome was assembled into 1377 scaffolds spanning 547.76 Mb (N50 = 55.14 Mb, L50 = 5), with 94% comprised in nine chromosome‐scale scaffolds and 93.9% Benchmarking Universal Single‐Copy Orthologs genes identified as single copy and 3.4% as duplicated. A high degree of synteny, with minor and mostly telomeric rearrangements, was found when comparing this taxon with the previously reported genome of South American *C. pallidicaule* and the A‐subgenome chromosomes of *C. quinoa*. Phylogenetic analysis was performed using 10,588 single‐nucleotide polymorphisms generated by resequencing a panel of 41 New World AA diploid accessions and the Eurasian H‐genome diploid *Chenopodium vulvaria*, along with three AABB tetraploids previously sequenced. Phylogenetic analysis of these 32 taxa positioned the psammophyte *Chenopodium subglabrum* on the branch containing A‐genome sequences from the ATGC. We also present evidence for long‐range dispersal of *Chenopodium* diploids between North and South America.

## Core Ideas

- We report the genome assembly for a North American A‐genome *Chenopodium* diploid quinoa relative, *Chenopodium watsonii*.
- The genome of *C. watsonii* is similar to cultivated *Chenopodium pallidicaule* and the A genome of quinoa.
- Resequencing of 41 mostly North American diploids identified *Chenopodium subglabrum* as being closest to quinoa's A‐subgenome.

**Abbreviations**
ATGC: American tetraploid goosefoot complex
BUSCO: Benchmarking Universal Single‐Copy Orthologs
COG: cluster of orthologous genes
MLE: maximum likelihood estimation
NADH: non‐adhering pericarp
TE: transposable element

## 1 INTRODUCTION

Quinoa (*Chenopodium quinoa* Willd.) is an Andean‐origin pseudocereal having excellent seed protein for human consumption (Wu, 2015). Aside from this and its other nutritional benefits, such as high fiber, essential mineral content, and excellent fatty acid profile, quinoa is recognized for its tolerance to drought, salinity, and cold stresses (Azurita‐Silva et al., 2015; Bhargava & Srivastava, 2013; Biondi et al., 2015). On the other hand, quinoa's poor heat tolerance has presented an impediment to its successful introduction into lowland tropical and subtropical environments (Zurita‐Silva et al., 2014). Fortunately, quinoa produces mostly fertile hybrids when cross‐pollinated with its free‐living, heat‐tolerant North American (pitseed goosefoot, *Chenopodium berlandieri* Moq.) and South American (avian goosefoot, *Chenopodium hircinum* Schreb.) sister species, which together constitute the American tetraploid goosefoot complex (ATGC, 2*n* = 4*x* = 36, AABB subgenomes; Jellen et al., 2019; Wilson & Manhart, 1993). The ATGC also includes the Mesoamerican domesticated vegetable and pseudocereal forms of huauzontle (*C. berlandieri* Moq. Subsp. *Nuttaliae* (Saff.) H. D. Wilson & Heiser; Cepeda‐Cornejo et al., 2016; Wilson & Heiser, 1979).

Since the ATGC's AA and BB 18‐chromosome diploid ancestors are potential genetic resources for improving quinoa and huauzontle, their characterization is an important step in determining the tertiary gene pool for these cultivated species. At the diploid level, the ATGC's subgenome B is believed to only exist among Eurasian diploids *C. ficifolium* Sm., *C. suecicum* Murr., and *C. ucrainicum* Mosyakin & Mandák (Mandák et al., 2018; Mosyakin & Mandák, 2020; Walsh et al., 2015). Significantly, Subedi et al. (2021) recently reported on the potential of *C. ficifolium* as a model system for studying quinoa's molecular biology and physiology. In contrast, the A genome is found throughout the New World in a wide array of diploids that are adapted to mostly disturbed environments including highlands and subtropical to temperate steppes, deserts, alkaline basins, seashores, and forests (Table S1, Figure S1; Aellen, 1960; Aellen & Just, 1943; Benet‐Pierce & Simpson, 2010, 2014, 2017, 2019; Clemants & Mosyakin, 2004; Giusti, 1970; Mosyakin & Clemants, 1996; WCVP, 2022). This pattern of species radiation (Gavrilets & Losos, 2009; Schluter, 1996) in the AA diploids appears to be unusual within the genus *Chenopodium* and elucidation of the mechanism responsible for this variation invites further study.

Whole‐genome assemblies can serve as powerful resources for assessing phylogenetic relationships (Eisen & Fraser, 2003), allelic diversity (The 100 Tomato Genome Sequencing Consortium et al., 2014), domestication pathways (Xie et al., 2019), and evolution of chromosome structural variation (Kamal et al., 2022). Mangelson et al. (2019) reported a short read‐based, Hi‐C scaffolded whole‐genome assembly for the domesticated Andean A‐genome diploid *kañiw*a or *cañahua* (*Chenopodium pallidicaule* Aellen). Various phylogenic studies (Brown et al., 2015 ; Jarvis et al., 2017; Štorchová et al., 2015 ; Walsh et al., 2015), however, indicated that the North American AA diploids are a distinct clade relative to the AA diploids of South America (including *C. pallidicaule*). In order to provide a suitable reference genome that is more closely related to the broad group of North American AA diploids, we selected Watson's stinking goosefoot (*Chenopodium watsonii* A. Nelson) for whole‐genome sequencing. Watson's stinking goosefoot is a primarily autogamous annual forb native to the Upper Sonoran Zone, Colorado Plateau, and High Plains, particularly on high‐fertility, disturbed, semi‐arid sites. A preliminary analysis of read matches with quinoa A‐genome sequences (Jellen et al., 2019) revealed 99.31% similarity between this subgenome and *C. watsonii*, higher match values than were measured with quinoa versus *Chenopodium sonorense* (99.23%) or quinoa versus Andean *C. pallidicaule* (98.53%). In addition, Watson's stinking goosefoot is a well‐characterized taxonomic entity within an otherwise poorly characterized genus (Benet‐Pierce & Simpson, 2014, 2017, 2019). This whole‐genome assembly was then used to determine phylogenetic relationships within a panel of mostly North American AA diploid taxa but including several South American A‐genome diploid samples.

## 2 Materials and Methods

### 2.1 DNA collection and long‐read sequencing

The *C. watsonii* accession BYU 873 (Table S1, Figure S1), collected in rural Yavapai County, Arizona, was grown hydroponically in a growth chamber set to a photoperiod of 11 h with broad‐spectrum lighting. Temperature controls were set between 18 and 20°C. The hydroponics solution was made using 27 g of MaxiGrow Hydroponics Plant Food (General Hydroponics, Sevastopol, CA) dissolved in 16 L of deionized water. The hydroponics solution was replaced every 2 weeks.

Prior to extraction, the *C. watsonii* plant was dark treated for 72 h. Young leaf tissue was harvested and DNA extracted using a modified protocol from Oxford Nanopore Technologies (Oxford, UK), “High Molecular Weight gDNA Extraction from Spinach Leaves,” using the QIAGEN (Hilden, Germany) Genomic‐top 500/G kit (Supplement 1). Following extraction, DNA quality was analyzed using the Thermo Scientific (Waltham, MA) NanoDrop One Microvolume UV‐Vis Spectrophotometer to check 260/280 and 260/230 absorbance ratios and Invitrogen^™^ (Waltham, MA) Qubit 3 fluorometer to estimate DNA concentration. Non‐fragmented samples were prepared using the DNA Clean & Concentrator‐5 kit (ZYMO Research, Irvine, CA). The protocol included with the ZYMO kit was followed to produce fragmented samples. Long‐read library preparation was done using the SQK‐LSK109 kit from Oxford Nanopore Technologies with Quick T4 DNA Ligase (NEB, M2200L) and 1D Genomic DNA by the ligation MinION protocol (Oxford Nanopore Technologies). Long‐read sequencing of the *C. watsonii* genome was completed using R9 flow cells from Oxford Nanopore Technologies on the MinION sequencing machine. Short‐read sequencing was performed using the same DNA on an Illumina (San Diego, CA) HiSeq platform with 350‐bp libraries and 150‐bp paired‐end sequencing.

### 2.2 Whole‐genome assembly of *C. watsonii*

Long‐read sequence data quality was checked using MinIONQC (Lanfear et al., 2018). Nanopore reads were trimmed and filtered using NanoFilt (De Coster et al., 2018) using the following options: −q = 8, headcrop = 25, and −l = 2000. Adaptor sequences were trimmed using Porechop v.0.2.3 (Wick, 2017) with the verbosity option set to 2.

**Core Ideas**

- We report the genome assembly for a North American A‐genome *Chenopodium* diploid quinoa relative, *Chenopodium watsonii*.
- The genome of *C. watsonii* is similar to cultivated *Chenopodium pallidicaule* and the A genome of quinoa.
- Resequencing of 41 mostly North American diploids identified *Chenopodium subglabrum* as being closest to quinoa's A‐subgenome.

Illumina short‐read data was trimmed, removing remnant adapter sequences, using the ILLUMINACLIP option from Trimmomatic v0.39 (Bolger et al., 2014). The following options were used within the pipeline: leading and trailing set to 20 bp; a sliding window of 4:20; and a minimum length of 75 bp.

A preliminary genome (BioProject PRJNA922607; accession SRR23112393) was assembled using CANU v.1.8 (Koren et al., 2017). Celera Assembler NU (CANU) parameters were set with normal corMhapSensitivity, 40 corOutCoverage, and parallel ovsMethod. The CANU‐assembled genome was polished using two rounds of RACON (Vaser et al., 2017); the first round used the ONT long reads and the second round with trimmed Illumina reads. Phase Genomics (Seattle, WA) produced the scaffolded assembly using the polished CANU assembly and Hi‐C data from dark‐treated, liquid nitrogen flash‐frozen leaf tissue. Contaminant reads were identified and removed using BlobTools (Laetsch & Blaxter, 2017). Chloroplast and mitochondrial DNA was identified and removed using NCBI BLAST against the quinoa chloroplast and mitochondrial genomes (Maughan et al., 2019). The HiC‐based assembly is available under NCBI BioProject PRJNA922607 as accession SRR23112395.

### 2.3 Transcriptome assembly

Leaf, root, and stem tissues from the hydroponically grown *C. watsonii* plant, in addition to a whole seeding, were used for RNA extraction using Trizol (Invitrogen^™^) and QIAGEN RNeasy spin column per the manufacturers’ instructions. RNA from each tissue and the whole seedling were combined in equal parts to create a single bulk sample. Library preparation and transcriptome sequencing was completed using the PacBio (Menlo Park, CA) Iso‐Seq platform on the Sequel II instrument at the BYU DNA Sequencing Center (Provo, UT). cDNA was prepared from the total RNA using a NEBNext^®^ single cell/low input cDNA synthesis and amplification kit (E6421L) which uses a template switching method to generate full length cDNAs (New England BioLabs, Ipswich, MA). IsoSeq libraries were prepared from the cDNA according to standard protocols using the SMRTbell v3.0 library prep kit (Menlo Park, CA).

The transcriptome was assembled using the Iso‐Seq reads and the IsoSeq v3 pipeline from the PacBio SMRT^®^ Tools software. The Iso‐Seq reads were aligned to the Hi‐C scaffolded assembly using the pbmm2 pipeline, another tool from SMRT^®^ Tools. Lastly, the transcripts were collapsed using IsoSeq v3. Transcriptome data is available in NCBI BioProject PRJNA922607 as accession SRR23112394.

### 2.4 Repeat analysis and gene annotation

RepeatModeler2 v.2.0.1 (Flynn et al., 2020) identified novel repeats in the assembled genome and RepeatMasker v.4.1.2 (Smit et al., 2013) classified the identified repeats using the RepBase/RepeatMasker database. MAKER v.2.31.0 (Bowman et al., 2017; Holt & Yandell, 2011) was used to annotate the final assembly in conjunction with AUGUSTUS (Stanke & Morgenstern, 2005) ab initio gene predications, the uniprot_sprot database from UniProtKB, sugar beet (Dohm et al., 2012), and quinoa protein sequences from Jarvis et al. (2017) for expressed sequence tags (EST) and protein homology. Genome completeness of the Hi‐C scaffold assembly was estimated using Benchmarking Universal Single‐Copy Orthologs (BUSCO) v5 (Simão et al., 2015) and two orthologous gene sets, the Embryophyte (embryophyta_obd10) and Viridiplantae (viridiplantae_obd10). A Circos plot of the assembled genome was created using Circa (https://omgenomics.com/circa) with concentric ring diagram elements consisting of chromosome sizes, gene density, GC content, and repeat distributions. The consensus sequence for telomeric repeats in plants was identified as TTTAGGG (Richards & Ausubel, 1988). The positions of the telomeric repeats within the assembly were determined using BLAST, as previously described (Jarvis et al., 2017).

### 2.5 Genome comparison

Synteny plots comparing the coding sequences of diploid *C. watsonii* and *C. pallidicaule*, as well as allotetraploid quinoa, were generated using CoGe SynMap (https://genomevolution.org/coge). DAGchainer (Haas et al., 2004) output file from the *C. watsonii* versus *C. pallidicaule* SynMap with the MCScanX toolkit (Wang et al., 2012) generated a collinearity file which was visualized using SynVisio (Bandi & Gutwin, 2020).

### 2.6 Resequencing, variant detection, and phylogenetic analysis

Seeds from 41 A‐genome *Chenopodium* diploid accessions, one H‐genome diploid, and three AABB tetraploid accessions from the germplasm collection at BYU and from small collections in South America (Table S2) were sterilized with 10% bleach, manually scarified, and germinated on filter paper in 9 cm Petri plates. Samples were treated with 1 mL of 30 μM potassium nitrate, 1 mL of 100 ppm gibberellic acid, and sprayed with Hi‐Yield Captan 50 W Fungicide. Young leaf tissue from the established plants was collected, freeze‐dried, and DNA was extracted using a modified mini‐salts extraction protocol (Supplement 2) (Dellaporta et al., 1983; Todd & Vodkin, 1996). Quality control parameters for concentration (<300 ng/mL) and contamination (260/280 and 260/230 ≅ 2.0) were followed before sequencing. All DNA samples were sent to Novogene Corporation, Inc. (San Diego, CA) for Illuming NovaSeq 6000 whole‐genome sequencing with 10× coverage of 150‐bp paired‐end reads from a 500‐bp insert library. The three tetraploid samples were previously sequenced by Jarvis et al. (2017).

Raw data in File ASsembly Tool Q (FASTQ) files, approximately 500 Mb per accession, were trimmed using the ILLUMINACLIP option from Trimmomatic v0.39 (Bolger et al., 2014) with the same parameters as previously described. The trimmed reads were subsequently mapped to the *C. watsonii* reference genome using Minimap2 v2.17 (Li, 2018) with a minimum read coverage depth of two and a minimum allele frequency of 51%. The output SAM files were sorted, duplicate reads were removed using fixmate and markdup and filtered for quality MAPping Quality (MAPQ > 45) using SAMtools v1.9 (Li, 2009). The filtered SAM files were subsequently converted to BAM files using the view tool from SAMtools. All DNA sequence files are publicly available under NCBI BioProject PRJNA922607 as accessions SRR23112388–SRR23112435.

### 2.7 SNP calling and phylogenetic analyses

Single‐nucleotide polymorphisms (SNPs) were identified using the BAM files and InterSNP, a program within the BamBam v1.4 pipeline (Page et al., 2014) which produced a SimpleSNP file containing the SNP and genomic location for each of the accessions relative to the reference genome. Parameters for InterSNP included a minimum read coverage of 5, missing data <10%, and since these species are highly autogamous, heterozygous SNPs were excluded from downstream analyses. The SNPhylo v20160204 pipeline (Lee et al., 2014) was used to filter representative SNPs using 500,000 bp sliding windows. SNPs were removed that had >10% missing data, a minor allele frequency <15%, and subject to a linkage disequilibrium threshold equal to 0.3. The IQ‐TREE (Nguyen et al., 2015) pipeline in conjunction with the PHYLIP SNP dataset (Felsenstein, 1989) produced by SNPhylo was used to generate a phylogenetic tree based on maximum likelihood estimation (MLE) with a bootstrap of *n* = 1000 and correcting for ascertainment bias using the +ASC option. SplitsTree5 (Huson, 1998) and FigTree v1.4.4 (Rambaut, 2010) were used for tree visualization. Loci were also assumed to be homozygous, as these species are primarily autogamous.

A MLE phylogenetic tree was generated using 1600 single‐copy orthologous genes identified with BUSCO and subsequently concatenated into whole‐gene alignment super matrices for either complete genomes (if diploid) or the separated subgenomes (if allopolyploid) from whole‐genome assemblies of six species: three *Chenopodium* diploids (*C. pallidicaule, C. suecicum*, *C. watsonii*); two *Chenopodium* polyploids ([*Chenopodium formosanum* Koidz., BBCCDD genome composition; Jarvis et al., 2022] and *C. quinoa* [AABB genome composition; Jarvis et al., 2017]); and one *Atriplex* diploid (*Atriplex hortensis* L.; Hunt et al., 2020). The 1600 genes were aligned with MAFFT v7.490 (Katoh et al., 2002). ALISCORE (Misof & Misof, 2009) and AliCUT (Kueck, 2017) were used to remove regions of the alignments that were indistinguishable from random noise. The alignments were concatenated using the FASconCAT‐G v1.11 software (Kueck & Longo, 2014) and a tree generated using IQ‐TREE v2.1.3 (Nguyen et al., 2015) with *n* = 1000 bootstrap support, which was then visualized using FigTree v1.4.4 (Rambaut, 2010). To ensure that our phylogeny was not biased by incomplete lineage sorting, we also created a multi‐species coalescent tree by first estimating1600 individual gene trees using IQ‐TREE (v. 2.1.3), then using ASTRAL (v. 5.7.1) to estimate the species tree from this dataset.

## 3 RESULTS AND DISCUSSION

### 3.1 Whole‐genome assembly of *C. watsonii*

Oxford Nanopore long‐read sequencing produced 45.5 Gb of data included in 3.67 million reads, representing a genome coverage of ∼82×. The N50 from the sequencing reads was 13,761 bp with an average read length of 12,383 bp with a maximum read size of 190 kb and average quality score of 13. The primary contig assembly consisted of 3517 contigs with a total assembly length of 551.37 Mb. The contig N50 was 553.04 kb with an L50 of 231. The contig assembly contained 3520 gaps and 3533 Ns (Table 1). Five unincorporated contigs determined to be derived from contaminating insect DNA (Thysanoptera and Coleoptera) were identified using Blobtools and subsequently removed; one of these contigs spanned 29,766 bp while the others were less than 7000 bp. The Blobplot produced by Blobtools showed an average GC content of 37% with read coverage averaging around 80× (Figure S2).

**TABLE 1:** Assembly statistics for the *Chenopodium watsonii* primary contig and Hi‐C scaffold assemblies.

Assembly statistics	Primary	Hi‐C
Assembly size (Mb)	551.37	547.76
Number of contigs/scaffolds, respectively	3517	1377
N50 (Mb)	0.553	55.14
L50	231	5
Longest (Mb)	5.17	64.48
N count	3533	187907
Gaps	3520	5338
N90 (Mb)	0.062	53.65
L90	1399	9

**FIGURE 1:**
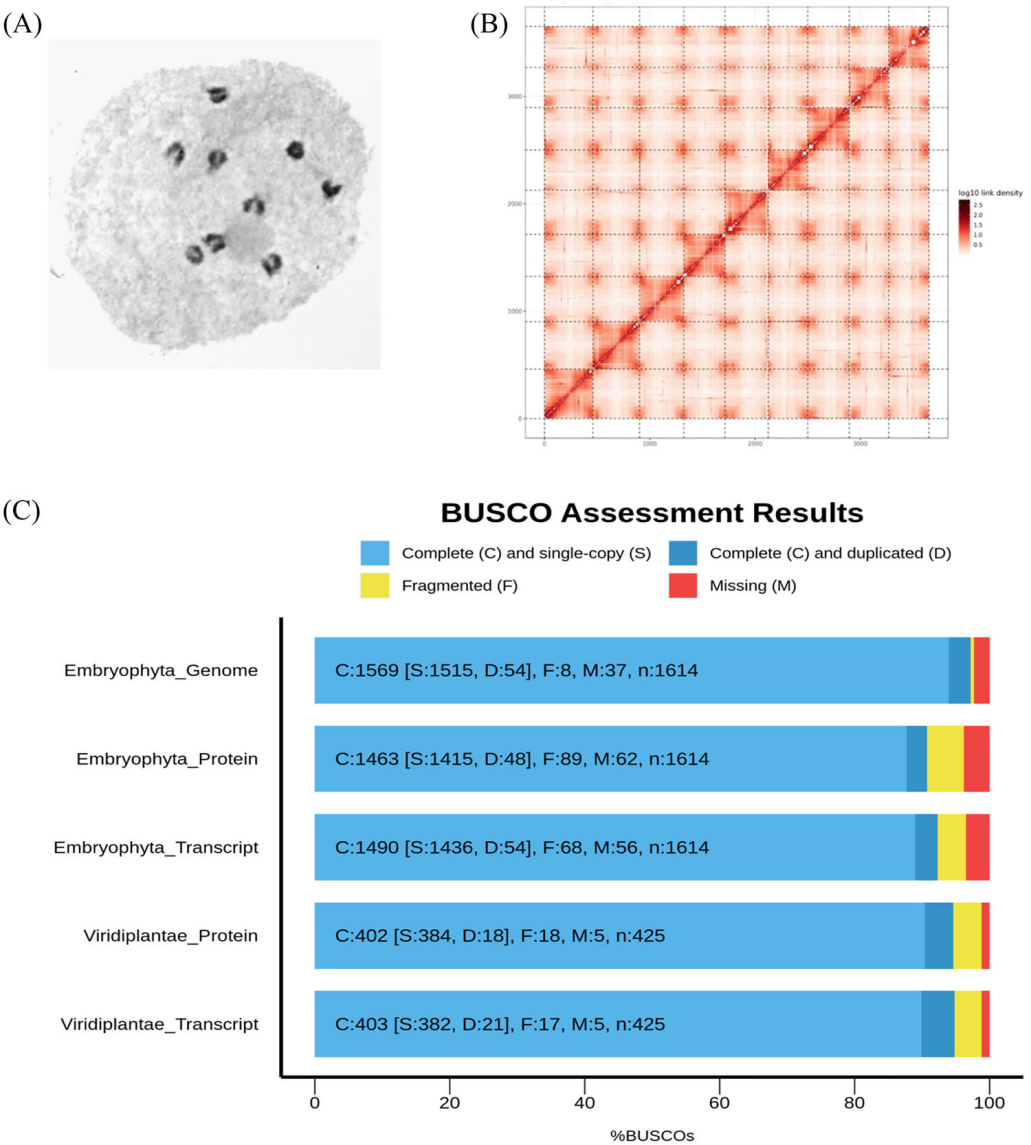
(A) *Chenopodium watsonii* chromosomes. Nine ring bivalents at diakinesis stage of prophase I. (B) Hi‐C linkage density heat map with nine distinct scaffolds. (C) BUSCO assembly statistics against the Embryophyta and Viridiplantae orthologous gene sets for the assembled genome, transcriptome, and protein annotation.

Chromatin‐contact mapping using 163 million read pairs (48.0 Gb) of 2 × 150 bp paired‐end Illumina Hi‐C data yielded nine chromosome‐length pseudomolecule scaffolds, representing 94% of the total contig sequence data. These pseudomolecules presumably correspond to the nine (*n* = 9) haploid chromosomes of *C. watsonii* (Figure 1). An additional 1368 contigs/scaffolds were also present in the final assembly. They represented <6% of the total, were small (averaged 24 kb), and had a high repeat content making them difficult to reliably place with Hi‐C data. The Hi‐C chromosome‐scale assembly produced a total genome length of 547.76 Mb and an N50 of 55.14 Mb with an L50 of 5 (Table 1). Each chromosome‐scale scaffold contained a range of clustered contigs from 171 to 240, representing a total of 1844 contigs (52.2%) from the primary assembly. Chromosome lengths varied from 64.46 to 54.81 Mb, with an average length of 57.14 Mbp. The N count was 188 kb with 5364 assembly gaps. All gaps introduced by scaffolding with the Hi‐C data consist of 100 Ns. Interestingly, while both the primary contig assembly (551.37 Mb) and Hi‐C pseudochromosome assembly (547.76 Mb) genome‐size estimates are concordant with each other, they are somewhat lower than the published flow cytometry‐based genome size estimate of 645.48 Mb (Mandák et al., 2016), presumably due to incomplete coverage of highly repetitive regions containing ribosomal and satellite repeats.

### 3.2 Repeat analysis

Repeat Modeler identified 942,183 repetitive sequences, comprising 60.4% (330 Mb) of the assembled genome. RepeatMasker categorized the repetitive sequences as follows: DNA transposons made up 6.7% of the repetitive sequences; of these, 1.7% were classified as long interspersed elements (LINEs) and 32.7% were classified as long terminal repeats (LTRs), specifically *Copia* (14.9%) and *Gypsy* (17.6%) retrotransposons. In contrast, the *C. pallidicaule* genome was only 8.5% *Copia* elements, a significant decrease that is possibly due to an even less complete assembly of the *C. pallidicaule* genome (Mangelson at al. et al., 2019), which was based on Illumina short‐read sequencing technology. We note that the *C. pallidicaule* assembly (452 Mb) was also substantially smaller than the *C. watsonii* genome assembly (551 Mb) presented here, suggestive that the use of long‐read technology in the *C. watsonii* assembly produced a more contiguous assembly. As mentioned above, Mandák et al. (2016) reported four AA diploids as having haploid genome sizes ranging from 597 to 637 Mb, along with the calculated size of the A‐subgenome of quinoa (524 Mb; Jarvis et al., 2017). Alternatively, it is possible that the *C. watsonii* genome has had an expansion of the LTR elements since the divergence of the two species, resulting in the observed discrepancy. Another salient component of the *C. watsonii* repeatome is the 16.9% of the genome that remains unclassified. The high percentage of unclassified repeats is not unexpected as there is little representation of the Amaranthaceae in the Repbase database, thus, these elements are potentially unique to the chenopods. Low‐complexity elements, including simple sequence repeats (SSRs), microsatellites, and rRNA, comprised an additional 2.2% of the genome (Table S3).

**FIGURE 2:**
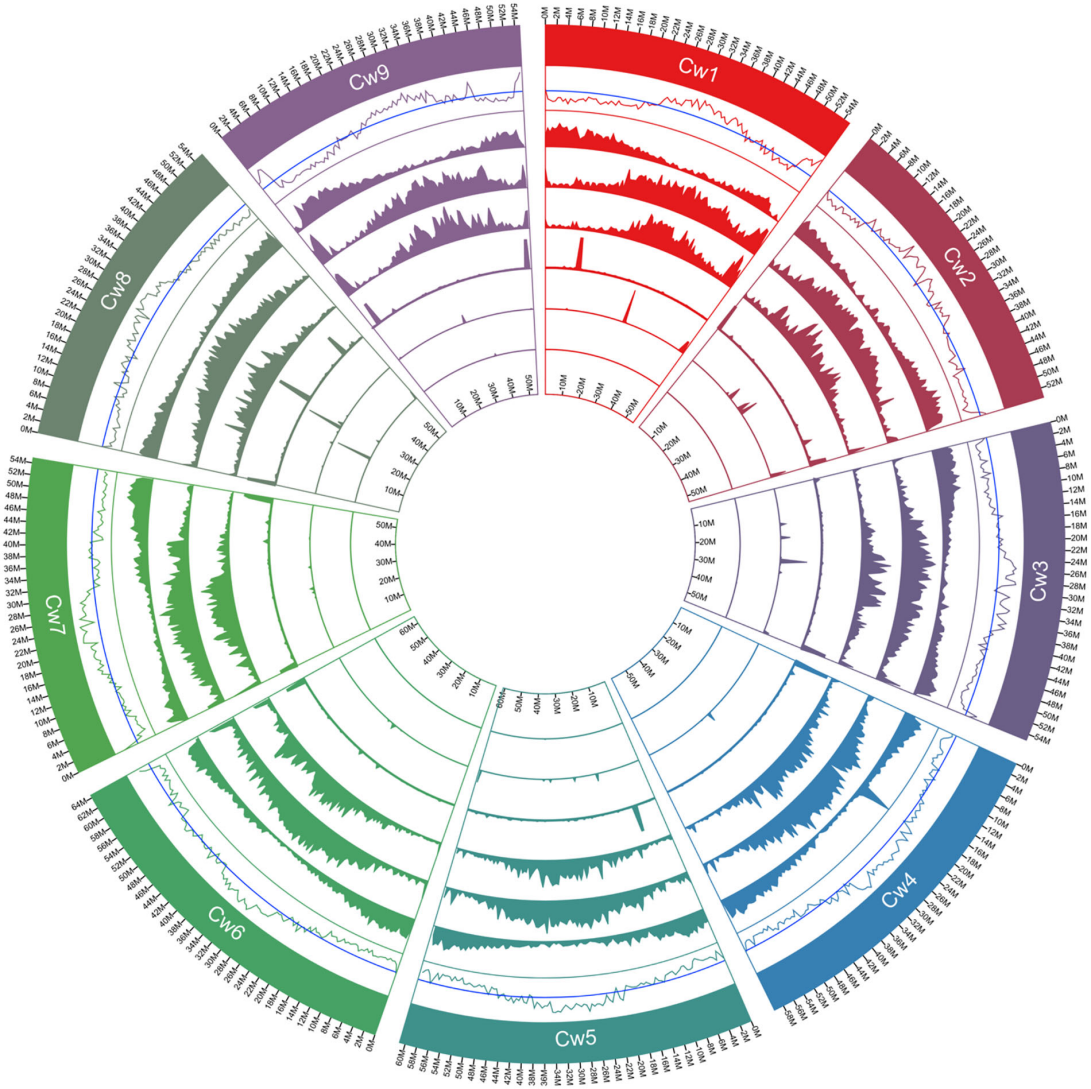
Genome overview of *Chenopodium watsonii* in 500 kb windows. Track 1 (outside): Chromosome and sizes; Track 2: GC content with mean (blue line = 37.3%; scale 33%–43%); Track 3: Annotated gene density; Track 4: LTR‐*Gypsy* TE distribution; Track 5: LTR‐*Copia* TE distribution; Track 6: Telomeric sub‐repeat distribution; Track 7: Centromere specific repeat (12‐13p; reference) density; Track 8: 5S rRNA gene distribution.

A spatial distribution of key genetic elements along the nine chromosomes of *C. watsonii* is provided in Figure 2. As expected, GC content (37.3%), *Gypsy* and *Copia* retroelement concentrations, and 12–13P centromeric repeats (Kolano et al., 2011) are elevated in the repeat‐rich, gene‐poor pericentromeric regions and are less abundant distally. Chromosomes 2, 3, 4, 6, 7, and 9 show clear peaks of telomeric sequence distributed at the ends of one or both arms (Richards & Ausubel, 1988). However, the telomeric sub‐repeat track apparently shows redistribution of these sequences interstitially on chromosomes 1, 5, 8, and possibly 9, with chromosome 8 having two telomeric interstitial peaks: one close to the centromere and the other farther out in the chromosome arm. The 5S rDNA sequence located using BLASTn is found on Cw8 and is consistent with the location on Cp8 in *C. pallidicaule* (Kolano et al., 2011; Mangelson et al., 2019).

### 3.3 Gene annotation

The MAKER program identified 30,725 gene models and 2254 tRNA genes. The average gene length was 3653 bp. Completeness was assessed using BUSCO with the Embryophyta and Viridiplantae BUSCO gene sets. The final assembly contained 1569 (97.2%) complete clusters of orthologous genes (COGs), which included 1515 (93.9%) single‐copy and 54 (3.3%) duplicated COGs with the embryophyta_obd10 set. Similarly, 419 (98.6%) complete COGs, including 396 (93.2%) single‐copy and 23 (5.4%) duplicated COGs were identified with the Viridiplantae_odb10 gene set (Figure S2). The low duplication rate is expected for a diploid species, while the high detection rate of complete single‐copy COGs is indicative of a high‐quality and complete genome. Annotation quality was assessed using annotation edit distance (AED) which considers specificity, sensitivity, and accuracy of the annotation. Eighty‐nine percent of the annotated genes had AED values <0.50 with an overall mean AED value of 0.23, suggesting a high‐quality annotation (Holt & Yandell, 2011).

### 3.4 Genome comparison and features

**FIGURE 3:**
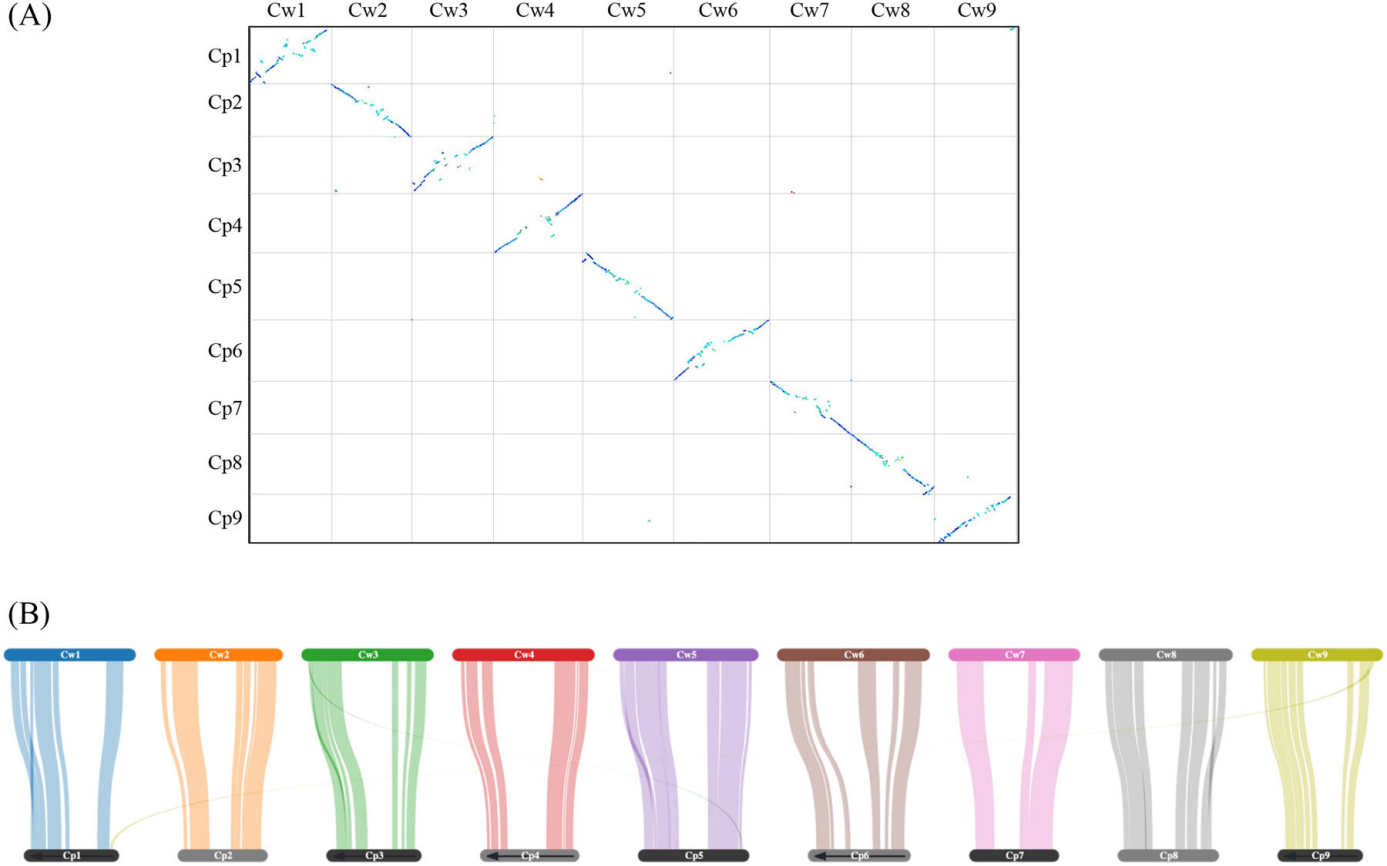
Comparison of *Chenopodium watsonii* and *Chenopodium pallidicaule* (Mangelsen et al., 2019) A‐genome diploid assemblies. (A) Synteny dot plot between *C. pallidicaule* (*y*‐axis, Cp) and *C. watsonii* (*x*‐axis, Cw); darker colors reflect high homology. (B) Ribbon plot comparing alignments of domesticated, Andean *C. pallidicaule* (bottom row, Cp) and wild, North American *C. watsonii* (top row, Cw) pseudochromosomes; synteny between Cp1‐9 and Cw1‐9, respectively.

**FIGURE 4:**
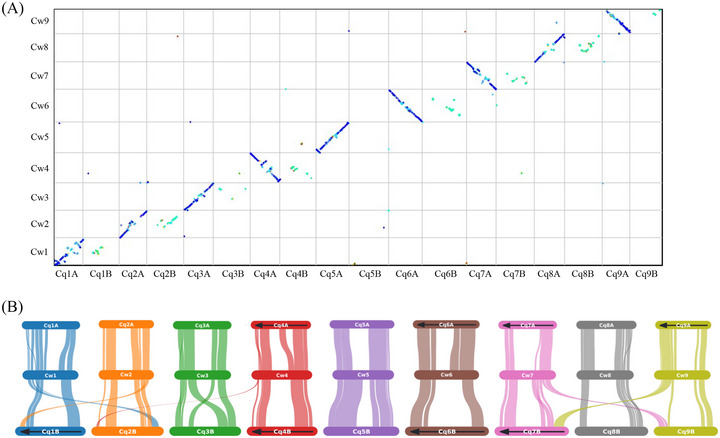
Comparison of *Chenopodium watsonii* with *Chenopodium quinoa* cv. “QQ74.” (A) Synteny dot plot between quinoa (*x*‐axis, Cq) and *C. watsonii* (*y*‐axis, Cw); darker dots represent high homology, almost exclusively with quinoa subgenome A. (B) Ribbon plot between quinoa (Cq) and *C. watsonii* (Cw) pseudochromosomes; synteny between Cq1A‐9A (top), Cw1‐9 (middle), and Cq 1B‐9B (bottom).

A high degree of synteny between *C. watsonii* and *C. pallidicaule* orthologous chromosomes was evident from the DAGChainer output generated by the SynMap (Haas et al., 2004), which included 16,521 syntenic coding sequences within 583 syntenic blocks, averaging 28 genes per block (Figure 3). Similarly high levels of synteny were observed between *C. watsonii* and the two subgenomes of *C. quinoa*, including 16,539 and 15,708 syntenic coding sequences shared with the A‐ and B‐subgenomes of *C. quinoa*, respectively (Figure 4). The larger number of shared syntenic coding sequences between the *C. watsonii* genome and the A‐subgenome of quinoa is likely reflective of shared ancestry. This shared ancestry is also observed in the larger syntenic block size shared between *C. watsonii* and the A‐subgenome of *C. quinoa*, where the average gene count within a block is 35 and 32, respectively, for the A‐ and B‐subgenome of *C. quinoa*.

A comparison of mapped interstitial telomeric sequences (Figure 2) with the synteny plots of the A‐genome diploid *C. pallidicaule* (Figure 3) suggests the potential of a subtelomeric paracentric inversion on Cw1 with telomeric inversions on Cw5 and Cw8 and a potential telomeric inversion on Cp3 that does not show up as an internal telomeric sequence on Cw3. However, we must caution that Mangelson et al. (2019) noted that their PGA2 assembly algorithm had difficulty in correctly placing the canonical telomeric repeat on the Illumina short‐reads assembled into *C. pallidicaule* pseudochromosomes. The comparison of *C. watsonii* with *C. quinoa* subgenome A (Figure 4) identified a potential chromosome 4A telomeric inversion in *C. quinoa* in addition to the 1, 5, and 8 inversions also observed with *C. pallidicaule*. Mechanisms besides inversion that lead to interstitial migration of telomeric sequences—a relatively common phenomenon in plants—include translocation, transposition, gene amplification, and so on (Maravilla et al., 2021). Interstitial telomeric inversions have previously been ascribed to chromosome instability in microsatellite‐enriched regions in yeast (Aksenova et al., 2013). We note that these putative rearrangements on chromosomes 1, 5, and 8 need to be verified as they could alternatively represent assembly (specifically scaffolding) errors.

### 3.5 Phylogenetic analysis of *Chenopodium* A‐genome diploids

A maximum likelihood phylogenetic tree of 41 New World A‐genome *Chenopodium* diploid accessions rooted by the Eurasian H‐genome outgroup *Chenopodium vulvaria* L. was generated using SNPs identified by comparing the 10× resequencing data with the *C. watsonii* reference (Figure S3). InterSNP called 1,010,399 SNPs across the mapped reads with a minimum requirement of <10% missing data. The final SNP dataset analyzed by IQ‐Tree consisted of 10,588 SNPs, with an average of 1176 SNPs per chromosome. This analysis yielded robust bootstrap values with 90% of nodes having values >95% and resolved the set of AA diploids into eight well supported, monophyletic subgroups (Figure S3).

**FIGURE 5:**
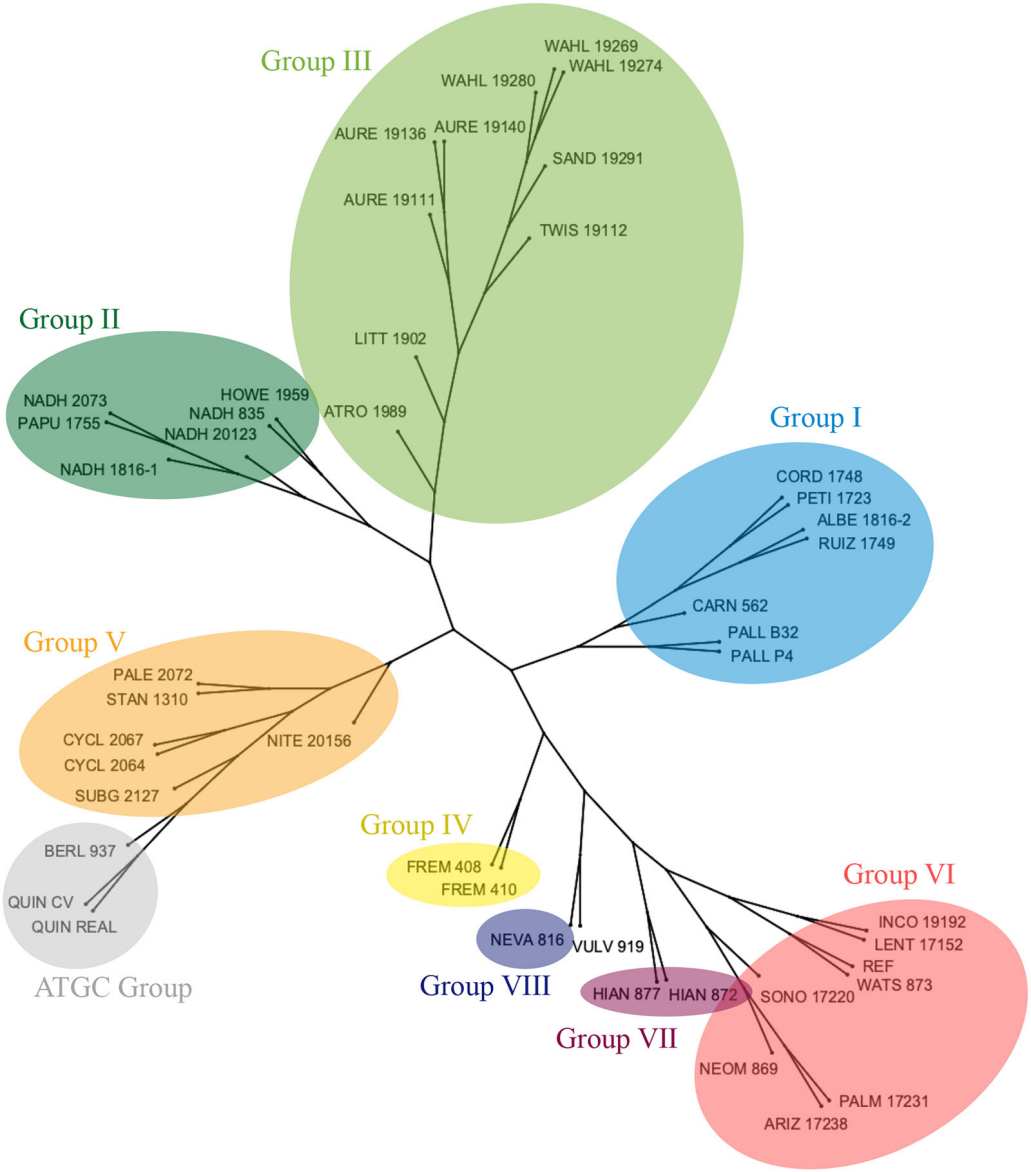
Midpoint‐rooted tree with colored clades and with *Chenopodium vulvaria* (VULV 919) as outgroup. The maximum likelihood tree was generated using SplitsTree and 10,588 SNPs after filtering using the following parameters: <10% missing data, minor allele frequency <15%, and linkage disequilibrium <30%. Bootstrap values are provided in Figure S3.

A second, midpoint‐rooted tree was also produced to investigate position of three members of the AABB species complex (two *C. quinoa* and one *C. berlandieri*) within the context of the A‐subgenome phylogenetic tree (Figure 5). In this analyses, 10× resequencing reads (Jarvis et al., 2017) that mapped specifically to the A‐subgenome of the *C. quinoa* reference were mapped, filtered, and called as previously described relative to the *C. watsonii* reference to produce genotypic calls at the same 10,588 SNP loci used in the previous phylogenetic analysis. As expected, this analysis largely reflected the previous tree, except for an additional species clade housing the A‐subgenomes of the three tetraploid accessions belonging to the ATGC (Figure 5). Below we systematically describe characteristics of the nine identified groups based on their order in the phylogenetic tree in Figure S3, from top to bottom. We note that while the goal was to survey all of the North American native AA taxa, we were unable to obtain samples for seven AA diploids identified in the scientific literature, namely *Chenopodium flabellifolium* Standl.; *Chenopodium foggii* Wahl; *Chenopodium incanum* (S.Wats.) Heller; *Chenopodium lineatum* Benet‐Pierce; *Chenopodium luteum* Benet‐Pierce*; Chenopodium parryi* Standl.; and *Chenopodium simpsonii* Benet‐Pierce. We also included two samples of *C. pallidicaule* and five other putative A‐genome diploids of South American origin to help provide perspective and context on geographic insularity of the North American species.

### 3.6 South American group (Group I)

Beginning at the top of the tree, all but one of the samples from South America formed a unified clade that was supported by a robust bootstrap value (>95%). As expected, domesticated Andean *C. pallidicaule* (PALL) samples grouped together and were closely related to a Pacific‐slope sample of *Chenopodium carnosolum* Moq. (BYU 562) from over 3100 m elevation on the Andean Cordillera Occidental of Tarapacá, Chile (Jarvis et al., 2017). Also grouping together were samples of *Chenopodium cordobense* Aellen (BYU 1748) and *Chenopodium petiolare* Kunth (BYU 1723) from Huascha, Córdoba Province, and Agua de las Palomas, Catamarca Province, Argentina, respectively. The other samples falling into this group were BYU 1816‐2, an accession of *Chenopodium albescens* Small from Laguna Salada in Brooks Co., Texas and a very similar sample of *Chenopodium ruiz‐lealii* Aellen (BYU 1749) from Chañar, La Rioja Province, Argentina.

### 3.7 Lejosperma–Leptophylla group 1 (Group II)

The next group consisted of five narrow‐leaved samples from North America and one from South America, designated for Aellen's Section *Chenopodia* Subsection *Lejosperma* that grouped strict‐sense *Chenopodium* taxa having narrow leaves, mostly smooth testas, and mostly non‐adhering (NADH) pericarps (Aellen & Just, 1943) and Mosyakin and Clemants’ (1996) designation of these species as subsection *Leptophylla*. All of the samples falling in this genetic group except for BYU 1959 (*Chenopodium howellii* Benet‐Pierce, from Adel, Oregon) have NADH and are morphologically similar to *Chenopodium leptophyllum* (Moq.) Nutt. ex S. Wats. In the case of *C. howellii* there is an adhering pericarp (achene) and rugose testa. Sample BYU 835 (*Chenopodium desiccatum* A. Nels. from Elko, Nevada) was closest to *C. howellii*. Overall plant morphology of these two taxa was very similar; however, the fruit of the former is a utricle while the latter has achenes. Accession BYU 20123 (*C. leptophyllum* from Colorado Springs) formed a group with BYU 1816‐1 (*Chenopodium pratericola* Rydb. from Brooks Co., Texas), *Chenopodium papulosum* Moq. (BYU 1755 from Matagusanos, San Juan Province, Argentina), and BYU 2073 (*C. pratericola* from Palo Pinto Co., Texas).

### 3.8 Atrovirens and “California Hians Aggregate” group (Group III)

Containing several species recently reclassified by Benet‐Pierce and Simpson (2019), this group of accessions included six taxa, all from California: *Chenopodium atrovirens* Rydb. (BYU 1989 from Monitor Pass, Alpine Co.); *Chenopodium littoreum* Benet‐Pierce & Simpson (BYU 1902, a prostrate psammophyte from coastal dunes in San Luis Obispo Co.); *Chenopodium aureum* Benet‐Pierce (BYU 19111, 19136, 19140, all from the Sierra Nevada Mountains); *Chenopodium twisselmannii* Benet‐Pierce (BYU 19112 from the Kern River Plateau); *Chenopodium sandersii* Benet‐Pierce (BYU 19291 from the San Gabriel Mountains); and *Chenopodium wahlii* Benet‐Pierce (BYU 19269, 19274, 19280, all from the Peninsular Ranges of Riverside Co. and San Diego Co.). The fruits of *C. atrovirens* are the only ones of this group having utricles rather than achenes.

IThere were several plant morphological discrepancies that should be noted within this group. First, plants of BYU 19269 (Supplemental Figure S1I) and 19280 were relatively tall (>5dm) and very weakly branched laterally and were therefore initially classified as *C. simpsonii* Benet‐Pierce. In contrast, BYU 19274 (Supplemental Figure S1J) perfectly fit the description of *C. wahlii* in the taxonomic key of Benet‐Pierce and Simpson (2019), but its fruits more closely fit the description of *C. simpsonii*.

### 3.9 Fremontii group (Group IV)

The two samples *of Chenopodium fremontii* S. Wats., BYU 408 (San Gabriel Mts., CA) and BYU 410 (Sierra Nevada Mts., CA) grouped together into a single clade. This taxon has large, warty to smooth seeds that are NADH and have broad triangular to ovate leaves with a distinct, earthy odor due to the presence of geosmin.

### 3.10 Lejosperma–Leptophylla group 2 (Group V)

This group encompasses five diploid taxa, four of which were classified previously in subsections *Lejosperma* or *Leptophylla*: *Chenopodium cycloides* A. Nels.*, Chenopodium nitens* Benet‐Pierce & Simpson*, Chenopodium pallescens* Stand.*, Chenopodium standleyanum* Aell., and *Chenopodium subglabrum* (S. Wats) A. Nels. Our samples of *C. cycloides* (BYU 2064 and 2067) were collected in the gypsiferous sand hills and along disturbed roadsides of the Permian Basin of West Texas. The taxon *C. pallescens* (BYU 2072, Eastland Co., Texas) is an episodic and apparently declining species that used to be widespread on disturbed, sandy tallgrass prairie (typical vegetation, *Andropogon gerardii*) soils from Northeast Texas and through Oklahoma, Eastern Kansas, Missouri, Eastern Nebraska, Iowa, and Southern Illinois. The psammophytic species *C. subglabrum* (BYU 2127, Seminoe Sand Dunes, Wyoming) is characterized by having very narrow leaves which range from fleshy to non‐fleshy and are minimally farinose. The testa of the characteristically large seeds (∼1.5 mm) ranges from rugose to pitted with an adhering pericarp. Our sample of *C. standleyanum* (BYU 1310, Scott Co., Missouri) is from sandy oak‐hickory woodlands of central North America. In contrast to these Great Plains species, *C. nitens* (BYU 20156, Mogollon Plateau, Arizona) characteristically grows on dry volcanic lake beds in *Pinus ponderosa* forests of Western North America.

The midpoint‐rooted tree (Figure 5) includes three samples of AABB tetraploids, which group on a distal branch within Group V. Accession BYU 2127 occupies a position at the base of this branch and therefore must be considered the best candidate donor of subgenome A in the *C. berlandieri–quinoa* allotetraploid group.

### 3.11 *Cellulata*–*Favosa* group (Group VI)

Aellen and Just (1943) assigned alveolate, honeycombed, achene‐fruited species to Sect. *Chenopodia* Subsect. *Cellulata* while Mosyakin and Clemants (1996) designated these as subsect. *Favosa*. This group of taxa similar morphologically to *Chenopodium neomexicanum* Stand. was expanded by Benet‐Pierce and Simpson (2017) included seven diploid species: *Chenopodium arizonicum* Stand. (BYU 17238 from Arivaca, Arizona)*; Chenopodium lenticulare* Aell. (BYU 17152 from the Davis Mts. in West Texas)*; C. neomexicanum* (BYU 869 from Coconino Co., Arizona)*, Chenopodium palmeri* Stand. (BYU 17231 from Arivaca, Arizona)*, C. sonorense* Benet‐Pierce & Simpson (BYU 17220 from Tubac, Arizona), and *C. watsonii* (BYU 873 from Yavapai Co., Arizona). All of the samples in this group have the characteristic alveolate fruit with adhering pericarps (achenes) and leaves ranging from broadly elliptic to campanulate. Because of their similar fruit morphology to the AABB tetraploids and a lack of developmental abnormalities in quinoa × *C. neomexicanum* triploid hybrids, Wilson (1980) suggested the *Cellulata* diploid group as most likely containing the AA ancestor of the AABB tetraploids. However, as noted above these tetraploids did not fall out in Group VI with the diploid *Cellulata* species.

### 3.12 Hians group (Group VII)

This group consisted of two samples of *Chenopodium hians* Stand.: BYU 872 (Yavapai Co., Arizona) and BYU 877 (Catron Co., New Mexico). This species is found mostly in mountainous terrain of the Southwestern United States in and around the Colorado Plateau and is characterized by narrow, farinose, fleshy leaves. The fruits vary in appearance, having smooth testa and adhering to semi‐adhering pericarps that are alveolate.

### 3.13 Nevadense group (Group VIII)

*Chenopodium nevadense* Stand. grouped by itself as a basal position in the tree. Found mainly in the sodic clay pans of the Western Great Basin and valleys of the Eastern Sierra Nevada Mountains, *C. nevadense* is a highly episodic taxon having fleshy, farinose leaves that are rhombate to ovate in shape. The adherent pericarp is papillate and typically a pale white color (Standley, 1916). The sample included here, BYU 816, was collected on the Soda Lakes Playa in Churchill Co., Nevada.

### 3.14 Homoplasy in AA *Chenopodium* species

It is interesting to note that several key morphological characters appear in multiple clades, presumably due to convergent evolution. One obvious trait that apparently evolved in at least two lineages is narrow versus broad leaf blades, possibly in response to hydric stress and/or as an adaptation to sandy soils (Subbarao et al., 1995) since all of the narrow‐leaved species are either obligate psammophytes (e.g., *C. cycloides*, *C. littoreum*, *C. pallescens*, *C. subglabrum*, and the NADH taxa *C. desiccatum, C. leptophyllum* and *C. pratericola*) or are adapted to seasonally dry mountains (e.g., the *C. hians* complex) and playas (*C. nevadense* and *C. nitens*). While all taxa in Groups II, III, V, VII, and VIII have narrow leaves, all the others in Groups I, IV, and VI have broad leaf blades. The fact that all the taxa in Group V, which is most closely allied to the ATGC, have narrow leaves while all of the AABB tetraploids are broad leaved suggests that broad, toothed leaves might have been contributed by the B‐genome ancestor, a rational assumption given that all three extant B‐genome diploid species—*C. ficifolium*, *C. suecicum*, and *C. ucrainicum*—also have broad, toothed leaves.

*Chenopodium* taxonomists have long considered the pericarp (fruit wall) as a paramount morphological trait, with species delineated into adhering (achene), semi‐adhering, and non‐adhering (utricle) forms (Benet‐Pierce & Simpson, 2014; Mosyakin & Clemants, 1996). The *Lejosperma* and *Cellulata* subsections proposed by Aellen and Just (1943) divided species based on seed coat texture and pericarp adherence, with *Lejosperma* housing taxa with smooth to wavy seed coats and NADH and *Cellulata* housing taxa with pitted seeds and adhering pericarps. Mosyakin and Clemants (1996) further divided subsection *Lejosperma* into *Leptophylla, Chenopodium, Fremontiana*, and *Standleyana* based on additional morphological characteristics including leaf, seed, and plant morphology. In our phylogenetic analysis, however, pericarp morphology was homoplastic. In Group I, all samples except BYU 562 had NADH. In Group II, the same was true for all samples except BYU 1959. In Group III, BYU 1989 and BYU 1902 were the only samples with NADH although BYU 19291 and possibly BYU 19112 have semi‐adhering pericarps (Benet‐Pierce & Simpson, 2019). Group V was a mixture of adhering (*C. cycloides* and *C. pallescens*) and NADH (BYU 20156, BYU 1310, and BYU 2127) samples. The Group IV *C. fremontii* samples were both NADH while all the samples in Groups VI, VII, and VIII had adhering pericarps. Wentland (1965) hypothesized that the adhering pericarp trait in *Chenopodium album* L. might be associated with enhanced seed dormancy. If this is supposition is true, and our data indicate this trait varies in multiple AA‐diploid lineages, then pericarp adherence would have been under strong selective pressure and its historic consideration by taxonomists as a key species‐delineation trait should be reconsidered. However, after working with thousands of free‐living samples, we have never observed an association between pericarp adherence and dormancy, either in Eurasian or North American native *Chenopodium* accessions.

### 3.15 North–south reciprocal long‐range dispersal

The grouping of an Argentine Pampa sample of *C. papulosum* (BYU 1755) with Group II from North America indicates the potential for an ancient north–south intercontinental dispersal event. Similarly, the placement of the Texas endemic species *C. albescens* (BYU 1816‐2) squarely amid the South American Group containing *C. cordobense* (BYU 1748)*, C. petiolare* (BYU 1723), the *C. pallidicaule* samples, and *C. ruiz‐lealii* (BYU 1749) suggests a reciprocal south–north dispersal between Texas and the South American Pampas. On an April 2018 seed collection expedition to South Texas, our group found 10 populations of *C. albescens* spread across Brooks, Dimmit, Duval, Jim Hogg, Karnes, La Salle, and Webb Counties, indicating this is a well‐established species between San Antonio and the Rio Grande Valley (Jellen et al., 2019). Cruden (1966) provided an overview of seed dispersal via avian migration, postulating that bird populations carry seeds, stuck to the mud on wings and feet, by “mountain‐hopping” to and from South American via Central America. Additionally, in controlled experiments involving seed feeding, fecal recovery, and germination, Wongsriphuek et al. (2008) demonstrated that ducks are capable of dispersing viable *Chenopodium* seeds via endozoochory. These data suggest that migrating birds following the Central Flyway could have carried *Chenopodium* seeds back and forth between the temperate climates of North and South America at some point, or repeatedly, in antiquity (Cain et al., 2000).

### 3.16 Gene‐based tree analysis

Using all current whole‐genome *Chenopodium* assemblies, the COG‐based tree (Figure S4) showed distinct groupings of the four different *Chenopodium* subgenomes with *A. hortensis* L. (Hunt et al., 2020) as the outgroup. IQ‐Tree and an input matrix of 618,448 sites, including 28,912 parsimony informative and 73,787 singleton sites from within 1600 single‐copy orthologous genes identified with BUSCO from the embryphyta_obd10 gene set, generated a high‐quality tree backed up by 100% bootstrap support across all nodes. A COG‐based analysis allows for the inference of relationships based on evolutionary time, data that cannot be inferred from a SNP‐based phylogeny. Based on the assumption that all genes evolve similarly, gene‐based trees do not consider hybridization, gene conversion, or gene transfer (Boussau & Scornavacca, 2020; Heath et al., 2008). Within the three B‐genome accessions, the B‐subgenome of the Taiwanese species *C. formosanum* Koidz. falls closest to the Eurasian B‐genome diploid, *C. suecicum*, with the B‐subgenome of quinoa being at the basal position of the B‐genome group. The C‐subgenome of *C. formosanum* is the closest relative to the B‐genome group, followed by the D‐subgenome of *C. formosanum*. The A‐genome accessions form a separate group from the other subgenomes, with A‐diploid *C. pallidicaule* neighboring the A‐subgenome of quinoa and *C. watsonii* rooting the A‐genome group (Figure S4). The future assembly and analysis of high‐quality genome sequences from North and South American AA *Chenopodium* diploids will clarify radiational speciation mechanisms and shed further light on the origin of the economically important AABB species.

### 3.17 Conservation considerations

At least six of the taxa included in this study—*C. cycloides*, *C. howellii*, *C. lenticulare*, *C. littoreum*, *C. pallescens*, and *C. subglabrum*—appear to be declining in habitat when their current prevalence is compared with herbarium collections. Of these, the most critically imperiled taxon is coastal Californian *C. littoreum* (NatureServe.org, 2023). *C. cycloides, C. pallescens*, and *C. subglabrum* are listed as vulnerable to critically imperiled across their Western North American native ranges (NatureServe.org, 2023). Another species not included in this study, *C. foggii*, is a rare New England native that is listed as threatened in Maine and endangered in Massachusetts and New Hampshire by the Native Plant Trust (2023) and is imperiled to critically imperiled according to NatureServe.org (2023). These species should be added to the list of threatened North American crop wild relatives compiled by Khoury et al. (2020) and targeted for enhanced conservation efforts, as the Canadian government has done for *C. subglabrum* (COSEWIC, 2006).

## AUTHOR CONTRIBUTIONS

**Lauren A. Young**: Conceptualization; data curation; formal analysis; investigation; methodology; writing—original draft. **Peter Jeffrey Maughan**: Conceptualization; data curation; investigation; methodology; resources; validation; writing—review and editing. **David E. Jarvis**: Conceptualization; data curation; funding acquisition; investigation; methodology; visualization; writing—review and editing. **Spencer P. Hunt**: Formal analysis; investigation; methodology; writing—review and editing. **Heather C. Warner**: Data curation; formal analysis; investigation. **Kristin K. Durrant**: Formal analysis; investigation. **Tyler Kohlert**: Investigation. **Ramiro N. Curti**: Investigation; resources; validation; writing—review and editing. **Daniel Bertero**: Conceptualization; formal analysis; investigation; resources; validation; writing—review and editing. **Gabrielle A. Filippi**: Data curation; formal analysis; investigation; resources; validation. **Tereza Pospíšilíková**: Investigation; resources. **Karol Krak**: Conceptualization; data curation; funding acquisition; investigation; methodology; resources; supervision; validation; writing—review and editing. **Bohumil Mandák**: Conceptualization; data curation; formal analysis; funding acquisition; investigation; methodology; project administration; resources; supervision; validation; writing—review and editing. **Eric N. Jellen**: Conceptualization; data curation; funding acquisition; investigation; project administration; resources; supervision; writing—original draft; writing—review and editing.

## CONFLICT OF INTEREST STATEMENT

The authors declare no conflict of interest.

## Supporting information

Supplemental Figure S1. Morphological diversity for fruits of *Chenopodium* AA diploids included in this study.

Supplemental Figure S2. Blobplot of read statistics in terms of coverage and GC content.

Supplemental Figure S3. A MLE‐based *C. vulvaria‐*rooted (VULV 919) tree of A‐genome diploid samples.

Supplemental Figure S4. Gene‐based tree including four *Chenopodium* sub‐genomes (A‐D) from five *Chenopodium* species and *Atriplex hortensis* generated using 1,600 COGs.

Supplemental Table S1. Current AA‐diploid *Chenopodium* taxonomy.

Supplemental Table S2. Resequencing panel providing accession names, BYU Welsh Herbarium (BRY) identifiers, NCBI (BioProject PRJNA922607) accession identifier, species, origin, collection coordinates, and figure labels in this paper.

## Data Availability

The *Chenopodium watsonii* genome for BYU 873 is publicly available online at CoGe (vV2.2, id63603; https://genomevolution.org/coge/). All of the sequencing data is publicly available under NCBI BioProject PRJNA922607 as accessions SRR23112388–SRR23112435 (BioSamples SAMN32670465–SAMN32670509).
