# Supplementary material for: A chromosome‐scale reference of Chenopodium watsonii helps elucidate relationships within the North American A‐genome Chenopodium species and with quinoa
Source: Plant Genome. 2023 May 17;16(3):e20349. doi: 10.1002/tpg2.20349 (PMC12807170; doi:10.1002/tpg2.20349)
Supplement: Supplementary file 1 [file TPG2-16-e20349-s001.docx]

Supplemental Table S2. Resequencing panel providing accession names, BYU Welsh Herbarium (BRY) identifiers, NCBI (BioProject PRJNA922607) accession identifier, species, origin, collection coordinates, and figure labels in this paper.

| **Accession Name** | **BRY** | **NCBI Accession** | **Species** | **Origin** | **Coordinates (Lat, Long)** | **Figure Label ID** |
| --- | --- | --- | --- | --- | --- | --- |
| BYU 1816-2 | 194530 | SRR23112435 | *C. albescens* | Texas | 27.1569, -98.07140 | ALBE 1816-2 |
| BYU 17238 | 190615 | SRR23112434 | *C. arizonicum* | N America | 31.5897, -111.32100 | ARIZ 17238 |
| BYU 1989 | 190627 | SRR23112423 | *C. atrovirens* | N America | 38.6717, -119.58686 | ATRO 1989 |
| BYU 19111 | 190622 | SRR23112412 | *C. aureum* | N America | 36.047496, -118.19806 | AURE 19111 |
| BYU 19136 | --- | SRR23112401 | *C. aureum* | N America | 37.56043, -118.67159 | AURE 19136 |
| BYU 19140 | --- | SRR23112392 | *C. aureum* | N America | 37.53527, -118.70678 | AURE 19140 |
| BYU 937 | 194513 | SRR4300216 | *C. berlandieri* | N America | 29.30524, -94.90580 | BERL 937 |
| BYU 562 | --- | SRR23112390 | *C. carnosulum* | Chile | -19.74031, -69.24114 | CARN 562 |
| BYU 1748 | --- | SRR23112389 | *C. cordobense* | Argentina | -30.5828, -64.73270 | CORD 1748 |
| BYU 2064 | 190624 | SRR23112388 | *C. cycloides* | Texas | 31.778813, -103.33882 | CYCL 2064 |
| BYU 2067 | 190621 | SRR23112433 | *C. cycloides* | Texas | 31.687546, -103.02716 | CYCL 2067 |
| BYU 408 | 190629 | SRR23112432 | *C. fremontii* | N America | 34.37963, -117.70712 | FREM 408 |
| BYU 410 | 190628 | SRR23112431 | *C. fremontii* | N America | 37.13243, -118.42768 | FREM 410 |
| BYU 872 | 195065 | SRR23112430 | *C. hians* | N America | 34.51477, -112.00698 | HIAN 872 |
| BYU 877 | --- | SRR23112429 | *C. hians* | N America | 34.19945, -108.93878 | HIAN 877 |
| BYU 1959 | 292616 | SRR23112428 | *C. howellii* | California | 42.205015, -120.01561 | HOWE 1959 |
| BYU 19192 | --- | SRR23112427 | *C. incognitum* | N America | 38.806676, -104.85103 | INCO 19192 |
| BYU 17152 | --- | SRR23112426 | *C. lenticulare* | Texas | 30.6911, -103.78910 | LENT 17152 |
| BYU 1902 | 190631 | SRR23112425 | *C. littoreum* | California | 35.0559, -120.60330 | LITT 1902 |
| BYU 1816-1 | 194529 | SRR23112424 | *C. pratericola* | N America | 27.1569, -98.07140 | NADH 1816-2 |
| BYU 20123 | 190619 | SRR23112422 | *C. pratericola* | N America | 38.799099, -104.73271 | NADH 20123 |
| BYU 2073 | 190626 | SRR23112421 | *C. pratericola* | N America | 32.4987205, -98.52292 | NADH 2073 |
| BYU 835 | 002313 | SRR23112420 | *C. desiccatum* | N America | 40.99821, -115.87011 | NADH 835 |
| BYU 869 | 194537 | SRR23112419 | *C. neomexicanum* | N America | 34.95468, -111.43585 | NEOM 869 |
| BYU 816 | 002335 | SRR23112418 | *C. nevadense* | N America | 39.51815, -118.88073 | NEVA 816 |
| BYU 20156 | 190632 | SRR23112417 | *C. nitens* | N America | 35.476333, -112.01179 | NITE 20156 |
| BYU 2072 | 190630 | SRR23112416 | *C. pallescens* | N America | 32.4987205, -98.52292 | PALE 2072 |
| PALL B32 | --- | SRR23112415 | *C. pallidicaule* | Bolivia | -16.67403, -68.31833 | PALL B32 |
| PALL P4 | --- | SRR23112414 | *C. pallidicaule* | Peru | -15.7693, -70.27050 | PALL P4, PALL 1785 |
| BYU 17231 | 195068 | SRR23112413 | *C. palmeri* | N America | 31.7733, -111.46600 | PALM 17231 |
| BYU 1755 | --- | SRR23112411 | *C. papulosum* | Argentina | -31.3342, -68.60660 | PAPU 1755 |
| BYU 1723 | --- | SRR23112410 | *C. petiolare* | Argentina | -27.6202, -66.12180 | PETI 1723 |
| BYU 1439 | --- | SRR23112409 | *C. quinoa* cv. Cherry Vanilla | Chile | NA | QUIN CV |
| BYU 1633 | --- | SRR23112408 | *C. quinoa* cv. Real-1 | Bolivia | NA | QUIN REAL |
| BYU 1749 | --- | SRR23112407 | *C. ruiz-lealii* | Argentina | -30.544, -65.95670 | RUIZ 1749 |
| BYU 19291 | --- | SRR23112406 | *C. sandersii* | California | 34.35966, -118.01100 | SAND 19291 |
| BYU 17220 | --- | SRR23112405 | *C. sonorense* | N America | 31.6104, -111.05120 | SONO 17220 |
| BYU 1310 | --- | SRR23112404 | *C. standleyanum* | N America | 37.0103, -89.61000 | STAN 1310 |
| BYU 2127 | 190620 | SRR23112403 | *C. subglabrum* | N America | 41.101168, -106.93817 | SUBG 2127 |
| BYU 19112 | 190625 | SRR23112402 | *C. twisselmannii* | California | 36.047496, -118.19806 | TWIS 19112 |
| BYU 919 | --- | SRR23112400 | *C. vulvaria* | Eurasia | NA | VULV 919 |
| BYU 19269 | 288717 | SRR23112399 | *C. wahlii* | California | 33.736208, -116.71421 | WAHL 19269 |
| BYU 19274 | 190618 | SRR23112398 | *C. wahlii* | California | 33.31221, -116.86220 | WAHL 19274 |
| BYU 19280 | 190623 | SRR23112397 | *C. wahlii* | California | 32.924204, -116.48198 | WAHL 19280 |
| BYU 873, REF | --- | SRR23112396 | *C. watsonii* | N America | 34.51477, -112.00698 | WATS 873 |
