# Supplementary material for: A chromosome‐scale reference of Chenopodium watsonii helps elucidate relationships within the North American A‐genome Chenopodium species and with quinoa
Source: Plant Genome. 2023 May 17;16(3):e20349. doi: 10.1002/tpg2.20349 (PMC12807170; doi:10.1002/tpg2.20349)
Supplement: Supplementary file 2 [file TPG2-16-e20349-s002.docx]

**SUPPLEMENTAL TABLES**

Supplemental Table S1. Current AA-diploid *Chenopodium* taxonomy. Subsection designations are from Mosyakin & Clemants (1996) and Clemants & Mosyakin (2004).

| **Species** | **Native Range** | **Species** | **Native Range** |
| --- | --- | --- | --- |
| *C. albescens* Small | Texas | *C. luteum* Benet-Pierce | California |
| *C. arizonicum* Standl. | Southwest North America | *C. neomexicanum* Standl. | Southwest North America |
| *C. atrovirens* Rydb. | Western North America | *C. nevadense* Standl. | Great Basin |
| *C. aureum* Benet-Pierce | California | *C. nitens* Benet-Pierce & Simpson | Western North America |
| *C. brandegeeae* Benet-Pierce | Southern California | *C. pallescens* Standl. | Southern Great Plains |
| *C. bryoniifolium* Bunge | Eurasia | *C. pallidicaule* Aellen | Peru-Boliva |
| *C. carnosolum* Moq. | Chile | *C. palmeri* Standl. | North America |
| *C. cordobense* Aellen | Argentina | *C. papulosum* Moq. | Argentina |
| *C. cycloides* A. Nels. | Texas | *C. parryi* Standl. | Mexico |
| *C. desiccatum* A. Nels. | Western North America | *C. petiolare* Kunth | Argentina |
| *C. eastwoodiae* Benet-Pierce | California | *C. philippianum* Aellen | Andean South America |
| *C. flabellifolium* Standl. | San Martin Island, Mexico | *C. pilcomayense* Aellen | Argentina |
| *C. foggii* Wahl. | Eastern North America | *C. pratericola* Rydb. | North America |
| *C. fremontii* S. Wats. | Western North America | *C. ruiz-lealii* Aellen | Argentina |
| *C. hians* Standl. | Western North America | *C. sandersii* Benet-Pierce | Southern California |
| *C. howellii* Benet-Pierce | Northern California | *C. scabricale* Speg. | Argentina |
| *C. incanum* (S. Wats.) Heller | Western North America | *C. sonorense* Benet-Pierce & Simpson | Southwest North America |
| *C. incognitum* Wahl | Southwest North America | *C. standleyanum* Aellen | Eastern North America |
| *C. lenticulare* Aellen | Western Texas | *C. subglabrum* A. Nels. | Great Plains |
| *C. leptophyllum* (Moq.) Nutt. ex S. Wats. | Western North America | *C. twisselmannii* Benet-Pierce | California |
| *C. lineatum* Benet-Pierce | California | *C. wahlli* Benet-Pierce | Southern California |
| *C. littoreum* Benet-Pierce & Simpson | Coastal California | *C. watsonii* A. Nels. | Western North America |
