# Supplementary material for: A chromosome‐scale reference of Chenopodium watsonii helps elucidate relationships within the North American A‐genome Chenopodium species and with quinoa
Source: Plant Genome. 2023 May 17;16(3):e20349. doi: 10.1002/tpg2.20349 (PMC12807170; doi:10.1002/tpg2.20349)
Supplement: Supplementary file 3 [file TPG2-16-e20349-s003.docx]

SUPPLEMENTAL FIGURES

Supplemental Figure S1. Morphological diversity for fruits of *Chenopodium* AA diploids included in this study. A. *C. cycloides* BYU 2067; B. *C. neomexicanum* BYU 869; C. *C. sonorense* BYU 17220; D. *C. fremontii* BYU 408; E. *C. pallescens* BYU 2072; F. *C. subglabrum* BYU 2127; G. *C. watsonii* BYU 873; H. *C. albescens* BYU 1816-2; I. *C. wahlii/simpsonii* BYU 19269; J. *C. wahlii* BYU 19274; K. *C. desiccatum* BYU 835; L. *C. hians* BYU 872; M. *C. standleyanum* BYU 1310; N. *C. lenticulare* BYU 17152; O. *C. palmeri* BYU 17231; P. *C. arizonicum* BYU 17238; Q. *C. pratericola* BYU 1816-1; R. *C. littoreum* BYU 1902; S. *C. howellii* BYU 1959; T. *C. atrovirens* BYU 1989; U. *C. aureum* or *lineatum* BYU 19111; V. *C. twisselmannii* BYU 19112; W. *C. aureum* BYU 19136; X. *C. incognitum* BYU 19192; Y. *C. sandersii* BYU 19291; Z. *C. nitens* BYU 20156.

A	B

C	D

E	F

G	H

I	J

K	L

M	N

O	p

Q	R

S	T

U	V

W	X

Y	Z
