# Supplementary material for: A chromosome‐scale reference of Chenopodium watsonii helps elucidate relationships within the North American A‐genome Chenopodium species and with quinoa
Source: Plant Genome. 2023 May 17;16(3):e20349. doi: 10.1002/tpg2.20349 (PMC12807170; doi:10.1002/tpg2.20349)
Supplement: Supplementary file 4 [file TPG2-16-e20349-s004.docx]

Supplemental Figure S2. Blobplot of read statistics in terms of coverage and GC content. Circles and dots represent chromosome-scale scaffolds and unscaffolded contigs with diameters scaled to sequence length and coloration based on BLASTn taxonomic annotation.
