# Supplementary material for: A chromosome‐scale reference of Chenopodium watsonii helps elucidate relationships within the North American A‐genome Chenopodium species and with quinoa
Source: Plant Genome. 2023 May 17;16(3):e20349. doi: 10.1002/tpg2.20349 (PMC12807170; doi:10.1002/tpg2.20349)
Supplement: Supplementary file 5 [file TPG2-16-e20349-s005.docx]

Supplemental Figure S4. Gene-based tree including four *Chenopodium* sub-genomes (A-D) from five *Chenopodium* species and *Atriplex hortensis* generated using 1,600 COGs. Bootstrap values were 100% for all groups. Each color denotes a different subgenome, as follows: AA, light blue; BB, red; CC, orange; DD, yellow; outgroup, dark blue.
