# Supplementary material for: A chromosome‐scale reference of Chenopodium watsonii helps elucidate relationships within the North American A‐genome Chenopodium species and with quinoa
Source: Plant Genome. 2023 May 17;16(3):e20349. doi: 10.1002/tpg2.20349 (PMC12807170; doi:10.1002/tpg2.20349)
Supplement: Supplementary file 6 [file TPG2-16-e20349-s006.docx]

Supplemental Figure S3. A MLE-based *C. vulvaria-*rooted (VULV 919) tree of A-genome diploid samples. Bootstrap values from IQ-Tree are indicated by colored nodes based on SH-aLRT support values. Passport information is in Supplemental Table S2. Each color denotes a different clade delineation.
